# Load-bearing aerobic exercise prior to injury moderates systemic immunosuppression response to fracture

**DOI:** 10.3389/fphys.2025.1587766

**Published:** 2025-09-11

**Authors:** Lia K. Strait, Dayne Dewan, Kylie E. Williams, Tyler Guyer, Nick J. Willett, Robert E. Guldberg

**Affiliations:** Department of Bioengineering, Phil and Penny Knight Campus for Accelerating Scientific Impact, University of Oregon, Eugene, OR, United States

**Keywords:** prehabilitation, exercise immunology, osteoimmunology, bone regeneration, preclinical

## Abstract

Bone fracture non-unions are common and often lead to costly revision surgeries, long-term patient pain and loss of function. Identifying fractures at-risk for non-union remains challenging due to an incomplete understanding of underlying mechanisms. Preclinical and clinical studies have shown that dysregulated immune responses are linked to impaired healing. These studies have also identified fracture characteristics, biologic factors, and lifestyle habits associated with a higher risk of poor healing. However, the impact of exercise history on the immune response to fracture remains underexplored. Load-bearing aerobic exercise is known to modulate properties of bone and systemic inflammation, suggesting that exercise history could influence post-fracture immune responses and healing outcomes. Using a rat treadmill exercise and femoral segmental defect model, this study sought to determine if regular exercise pre-fracture affects the systemic immune response and healing outcomes. We hypothesized that pre-fracture treadmill running would attenuate immunosuppressive mediators—shown previously to correlate with poor healing—and improve bone regeneration compared to sedentary controls. Subjects that exercised before fracture had decreased post-fracture circulating immunosuppressive myeloid-derived suppressor cells and pain sensitivity, however there was no significant effect of prehabilitation on bone repair volume, defect bridging rate, or biomechanical properties.

## 1 Introduction

Fractures are a common clinical occurrence, with the average American expected to experience two fractures during their lifetime (Amin et al., 2014). Most fractures heal endogenously through well-coordinated mechanical and biological signals in the regenerative niche (Marsell and Einhorn, 2011). Disruptions in this signaling can lead to delayed or incomplete fracture healing, known as non-union. Fracture non-union occurrence rates can exceed 13% depending on bone type, and have devastating consequences, presenting a significant challenge to both clinician and patient (Szczęsny et al., 2011; Smith et al., 2014). Diagnosis of non-unions remains challenging, in part due to an incomplete mechanistic understanding of poor bone healing (Rodríguez-Merchán, 2021). Preclinical and clinical studies have identified risk factors and comorbidities, such as osteoporosis and a dysregulated immune response, to be associated with poor healing outcomes (Baht et al., 2018; Vantucci et al., 2020; Cheng et al., 2021; Gorter et al., 2021). Yet, it remains unclear how healthy lifestyle choices, such as a history of regular exercise before the initial injury, may affect healing outcomes.

The main characteristic of immune dysregulation associated retrospectively with non-union is prolonged inflammation. However, the immunological profile that contributes to non-union is not fully understood and could be a promising target for potent new immunotherapies (Evans et al., 2024). Following a fracture, the immune system triggers an inflammatory response that results in an influx of reparative phagocytic and progenitor cells into the fracture site in addition to clearance of bacteria and debris, and release of pro-angiogenic mediators (Schmidt-Bleek et al., 2012; 2015). Gradually, across the first 2 weeks post-fracture in humans, the pro-inflammatory response shifts towards an anti-inflammatory state, governed by biological and physical cues (Maruyama et al., 2020). Persistent inflammation can occur due to a variety of causes including: infection (Wang et al., 2017), injury severity (Ertel et al., 1995), or mechanical instability (de Seny et al., 2016). Sustained inflammation can accentuate the anti-inflammatory response and disrupt the temporal balance of acute inflammation and resolution, as well as creating a hyperactive angiogenic process, which has been indicated to be counterproductive to healing (Claes et al., 2006). Other sustained immune dysregulation, such as immunosuppression, increases the risk for fracture non-union (Evans et al., 2024). It is well-established that exercise maintains healthy musculoskeletal tissue and can regulate systemic inflammation (Distefano and Goodpaster, 2018; Frodermann et al., 2019). While certain comorbidities, such as age and metabolic dysfunction, have been identified alongside prolonged inflammation, a patients exercise history has yet to be factored into predicting the inflammatory response and overall healing outcome in a data-driven manner.

When assessing a patient’s potential to heal, it is generally accepted that a young, physically active, healthy individual will recover better from a musculoskeletal injury (Hvid et al., 2014; Briggs et al., 2016). However, there is no rigorous scientific evidence demonstrating how regular exercise influences musculoskeletal healing outcomes, particularly in fracture healing. The loading portion of aerobic exercise is known to increase bone mineral density and strength, and beneficially alter the bone microarchitecture (Hong and Kim, 2018; Song, 2022), whilst the aerobic component of exercise has been shown to modulate the immune system by decreasing pro-inflammatory adipokines and increasing anti-inflammatory cytokines, potentially aiding in the crucial transition from a pro-inflammatory to an anti-inflammatory bone fracture environment (Gleeson, 2004; Campbell and Turner, 2018; Scheffer and Latini, 2020). This interaction suggests that a history of regular exercise prior to injury could serve as a key preventative measure to decrease non-union risk through maintaining bone integrity and regulating immune function prior to and during recovery of bone injury (Xie et al., 2019).

Our study sought to elucidate whether regular exercise prior to surgery, termed “prehabilitation”, would modulate inflammation and improve bone healing in a rat femoral defect model. We hypothesized that 4 weeks of treadmill running would increase bone volume, bone mineral density and functional properties while decreasing systemic inflammation prior to surgical induction of a femoral fracture. We further hypothesized that animals prehabilitated (exercised prior to fracture) would exhibit a more moderate inflammatory response after fracture and improved overall healing compared to sedentary (i.e., normal cage activity) controls. To investigate the effects of prehabilitation on bone healing, young female Wistar rats were subjected to 4 weeks of exercise training on a treadmill before undergoing surgery to create a 2 mm femoral defect stabilized with internal fixation. All subjects remained sedentary for the following 8 weeks, over which time bone regeneration was quantified longitudinally, and the systemic immune response was profiled. While we did not see significant differences in healing outcomes, we found that prehabilitation increased helper T cells, ameliorated longitudinal elevation of immunosuppressive myeloid-derived suppressor cells, and attenuated pain sensitivity.

## 2 Materials and methods

### 2.1 Exercise model

All animal care and experimental procedures were approved by the University of Oregon Institutional Animal Care and Use Committee (IACUC, 20-32) and carried out according to the guidelines. To investigate how a history of exercise influences healing outcomes following the onset of trauma, a treadmill running protocol was established to ensure equivalent exercise parameters between animals. Nine-week-old female Wistar rats (Envigo) were acclimatized to treadmills (NordicTrack, modified) for 10 days (5 days/week), with speed and duration gradually increasing from 8 m/min with 5-degree incline for 5 min until reaching final exercise conditions of 18 m/min with 5-degree inclination for 30 min/day (Table 1). This regimen corresponded to approximately 70%–75% VO2 max. Rats continued with this regimen 5 days/week for 2 weeks before surgical injury induction. Animals were weighed at the beginning and end of the exercise protocol and had access to food *ad libitum*.

**TABLE 1:** Pre-conditioning protocol to acclimate animals to treadmill running. Pre-training occurred 5 days/week for 2 weeks. Daily duration and maximum speed were increased daily in the intervals given in the description row until top speed was reached, and final exercise protocol of 2 min of warm up at moderate speed and 30 min of exercise at top speed was reached.

Parameters	Week 1 - Preconditioning					Week 2 - Preconditioning				
Day	1	2	3	4	5	6	7	8	9	10
Top Speed (m/min)	8	10	12	14	16	16	18	18	18	18
Duration (mins)	10	14	16	16	18	20	21	25	30	32
Degree Incline	5	5	5	5	5	5	5	5	5	5
Description (m/min | min)	2 | 3 5 | 2 8 | 5	2 | 2 5 | 2 8 | 2 10 | 8	5 | 2 7 | 2 8 | 2 12 | 10	5 | 2 8 | 2 12 | 4 14 | 8	8 | 2 12 | 4 16 | 12	8 | 2 12 | 2 16 | 16	8 | 2 16 | 4 18 | 15	8 | 2 14 | 3 18 | 20	8 | 2 14 | 3 18 | 25	10 | 1 16 | 1 18 | 30

### 2.2 Segmental defect procedure

As in previously described procedures, a unilateral 2 mm segmental defect was surgically created in the left femur (n = 7/group, Envigo) (Williams et al., 2024). The sample size was determined *a priori* with previous bone regeneration and immune data immune in our group, with an effect size of 2.5, an 80% power of detection and a level of significance of 0.05 (G*Power software). Anesthesia was induced and maintained using isoflurane (VetOne) inhalation. Following induction, but prior to surgical procedure, all animals were given a subcutaneous injection of 1.0 mg/mL sustained-release buprenorphine (Wildlife Pharmaceuticals) for analgesia. In brief, an anterolateral skin incision was made to the thigh to expose the muscle, which was then blunt dissected between the planes of quadricep and hamstring to expose the femur. The femur was then cleaned of any remaining tissue, permitting for the placement, and securing of a radiolucent ultra-high molecular weight polyethylene (UHMWPE) internal fixation plate (Cramer’s Precision Manufacturing) with four stainless steel screws (HilcoVision). Segmental 2 mm defects were created in the mid-diaphysis of the femur with an oscillating saw (Conmed). Following defect creation, the muscle was closed with 4–0 Vicryl suture, and the skin closed with a subcutaneous Monocyrl suture and wound clips. Incision sites were cleaned and coated in a paste of newskin liquid bandage (advantice Health) and crushed metronidazole antibiotic tablets (Zydus Lifesciences) to mitigate chewing. 48-h post-op, animals were given a bolus injection of standard buprenorphine and 2 mL of lactated ringers solution subcutaneously. Animals were weighed daily for 5 days post-op, and then once weekly until end of study. After 8 weeks, animals were euthanized via CO2 asphyxiation using the Euthanex- Smart Box EA 32000 (E-Z Anesthesia) set to an initial 53% CO2 flow rate of the chamber volume per minute, and then at a rate of 10 Lpm, in accordance with AVMA guidelines.

### 2.3 Radiography and μCT

**FIGURE 1:**
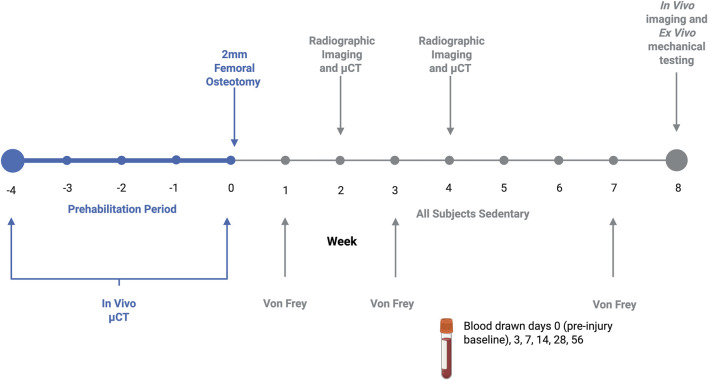
Graphical representation of experimental timeline. Exercising animals prehabilitated daily 5 days/week for 4 weeks, achieving a maximal exercise intensity of approximately 70%–75% VO2 max. Bone development in response to prehabilitation or sedentary conditions was quantified with micro CT. Following a 2 mm osteotomy fixed with a compliant internal fixation plate, all subjects remained sedentary after fracture. Throughout the 8-week recovery period, healing was assessed via micro CT and X-ray, with tactile allodynia quantified with Von Frey, and immune cells profiled through tail vein blood draws.

To assess bone adaptations to load-bearing exercise and examine pre-injury bone quality, *in vivo* imaging was performed before and after the 4-weeks of treadmill exercise. Bone was assessed qualitatively with two-dimensional digital radiographs and quantitatively with three-dimensional μCT scans. Following defect creation, longitudinal bone regeneration was assessed in the same manner at weeks 2, 4 and 8-post-op (Figure 1). All digital radiographs were taken at 40 kV with a 7 s exposure (Faxitron MX-20). Quantitative longitudinal bone regeneration was evaluated *in vivo* with the vivaCT80 (Scanco Medical) using a 48.5-μm voxel size, 55-kVp voltage, 145-μA current, and 750 ms integration time. Pre-operative volume of interest was defined by the central 1.5 mm of the mid-diaphysis of the femur. Post-operative volume of interest consisted of the central 1.5 mm of the 2 mm defect along the femoral axis. The “Bone Midshaft” evaluation script (Scanco Medical) was used to quantify polar moment of inertia (pMOI).

After 8 weeks, the animals were euthanized via CO2 asphyxiation. Left and right hind limbs were harvested for *ex vivo* μCT scanning. *Ex vivo* scans were performed in the same manner as *in vivo*, but with a 23.6-μm voxel size.

### 2.4 Biomechanical testing

After *ex vivo* μCT scanning, excised femora were wrapped in PBS-soaked gauze and stored at −20 °C overnight. The following day samples were thawed and remaining soft tissues outside of the defect were cleaned from the bone. Fixation plates were removed, and the native bone ends were potted in Wood’s metal. Potted femurs then underwent torsional testing to failure at a rate of 3°/s using the TA Electroforce 3200 series (TA Instruments) axial/torsion testing system. Maximum torque was quantified within the first 90-degrees of rotation. Torsional stiffness was calculated by finding the slope of the linear region before failure in the torque-rotation plot.

### 2.5 Von Frey

Sensitivity to stimulus was assessed at weeks 0 (baseline), 1, 3 and 7 as a means of indirectly measuring pain. Animals were placed into plastic boxes with a mesh floor, and they were then allowed 10 min to acclimate before an un-bending electronic Von Frey filament (BioSeb) was applied with linearly increasing force until the animal withdrew their paw. This was performed on both hindlimbs in triplicate and the paw withdrawal force recorded each time. To eliminate bias, the individual administering the stimulus was blinded and a second individual was responsible for placing the rats in the testing apparatus and recording force values.

### 2.6 Tissue collection and processing

To assess the longitudinal immune response, blood was drawn from the rat tail artery at days 0 (baseline), 3, 7, 14, 28 and 56 (Figure 1). 600 μL were collected with each draw, with 400 μL of whole blood collected into lithium heparin collection tubes (BD Microtainer™, Biosciences) and 200 μL collected in procoagulant tubes for serum isolation (Microvette™, Kent Scientific).

For whole blood processing, red blood cells were lysed using 1× RBC Lysis Buffer (eBioscience) according to the manufacturer’s instructions. Following lysis, cells were fixed using Flow Cytometry Fixation Buffer (R&D Systems), resuspended in FACS buffer containing 2% fetal bovine serum (FBS) in 1× phosphate-buffered saline (PBS), and stored at 4 °C until staining for flow cytometry.

### 2.7 Flow cytometry

Following processing, fixed whole blood samples were stained for flow cytometry analysis. To prevent nonspecific binding Fc receptors were blocked with purified mouse anti-rat CD32 (BD Biosciences) for 5 min at 4 °C before staining. A flow assisted cell sorting buffer (FACS Buffer) was made with 2% FBS in 1X PBS. Cells were then stained with anti-rat antibodies (eBioscience) at a dilution of 1:25 (antibody:FACS Buffer) for selected immune cell populations, including: T cells (CD3^+^), T helper cells (CD3^+^CD4^+^), cytotoxic T cells (CD3^+^CD8^+^), myeloid-derived suppressor cells (His48+CD11b/c+), B cells (B220+), monocytes (CD11b/c+, Novus, CD68^+^, Bio-Rad), M1 and M2 macrophages (CD68^+^CD11b/c+ and CD86^+^ or CD163+, respectively). Sample data were collected using a BD Accuri C6 flow cytometer and analyzed using FlowJo software. Gates were positioned based on fluorescence minus one controls with <1% noise allowed. Further details on antibodies can be found in Supplementary Table S1 and gating strategy in Supplementary Figure S2.

### 2.8 Statistical analyses

All data are reported as mean ± SEM. Longitudinal data were assessed for statistical significance (p < 0.05) using a repeated measures two-way ANOVA or mixed-effects analysis as appropriate, with multiple comparisons corrected for using Tukey’s or Sidak’s post-hoc tests, respectively. All statistical tests were executed using GraphPad Prism 10 software (Version 10.3.1 (464)). Sample sizes were determined by performing a power analysis in G*Power software based on bone volume and immune cell quantification results from previous studies. This *a priori* analysis determined a sample size of 6 was sufficient to draw statistically significant differences between groups. Post-hoc power analysis was also performed in G*Power software, and our actual power was found to be 0.9503.

## 3 Results

### 3.1 Load-bearing prehabilitation exercise stunted age-associated bone growth

**FIGURE 2:**
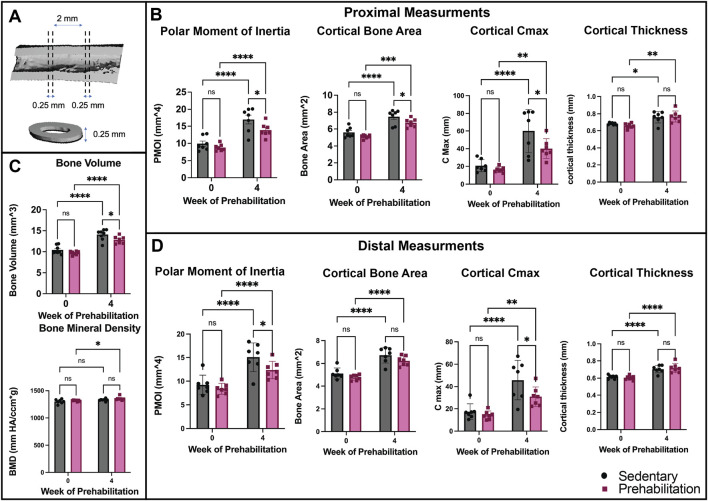
Exercise stunted age-associated bone growth **(A)** Representative femoral cross-section of intact bone indicating ROI used for quantification of bone volume and bone mineral density (2 mm region) and proximal and distal properties (0.25 mm on either end of mid-diaphysis). **(B)** Measurements of bone morphological properties of the proximal region of the bone. All groups experienced significant longitudinal increases. Exercising animals had a significantly lower cortical bone area (*p = 0.0339), cortical C max (*p = 0.0134) and polar moment of inertia (*p = 0.0186) post-exercise as compared to sedentary. **(C)** Bone volume and bone mineral density quantification within centered 1.5 mm of the femur mid-diaphysis prior to and after 1 month of treadmill running. Exercising animals had significantly lower bone volume compared to sedentary animals at time of surgery (*p = 0.0181). **(D)** Distal measurements of bone development. As in the proximal end, all groups exhibited significant longitudinal growth, but prehabilitation had reduced polar moment of inertia (*p = 0.0253) and cortical C max (*p = 0.0159) as compared to the sedentary at the end of 4 weeks of exercise. Data were analyzed with a repeated measures two-way ANOVA with multiple comparisons corrected for with Tukey’s post-hoc test (n = 7/group, p < 0.05, *p < 0.05, **p < 0.01, ***p < 0.001, ****p < 0.0001) and are displayed as mean ± sem.

Before the beginning of the prehabilitation protocol and following the 4-week treadmill training period, *in vivo* micro-computed tomography was used to assess initial bone properties and adaptations to exercise. As expected, due to age-associated bone growth, both prehabilitation and sedentary groups saw significant longitudinal increases in bone morphological properties of bone volume (Figure 2C), cortical thickness, cortical bone area, and polar moment of inertia (Figures 2B,D). However*,* we found that contrary to our hypothesis, prehabilitated animals had significantly lower bone volumes at the end of the exercise period as compared to the sedentary animals (Figures 2B,D). In fact, in addition to bone volume, pre-fracture cortical bone area, Cmax and polar moment of inertia were significantly lower in the prehabilitation group than in the sedentary subjects on the proximal end (Figure 2B). On the distal end, similar effects were observed with reduced polar moment of inertia and Cmax (Figure 2D) as compared to sedentary. Of interest, was the minor, but significant longitudinal increase in bone mineral density in the exercising group, but not sedentary (Figure 2D). At no timepoint were there any significant differences in body weight between groups, with no indication of overall stunted growth (Supplementary Figure S1). Overall, load-bearing aerobic prehabilitation exercise in young animals had a deleterious effect on bone morphological properties and left the prehabilitation group with lower cortical bone area and polar moment of inertia at the time of surgical bone defect creation.

### 3.2 Prehabilitation did not improve healing outcome

**FIGURE 3:**
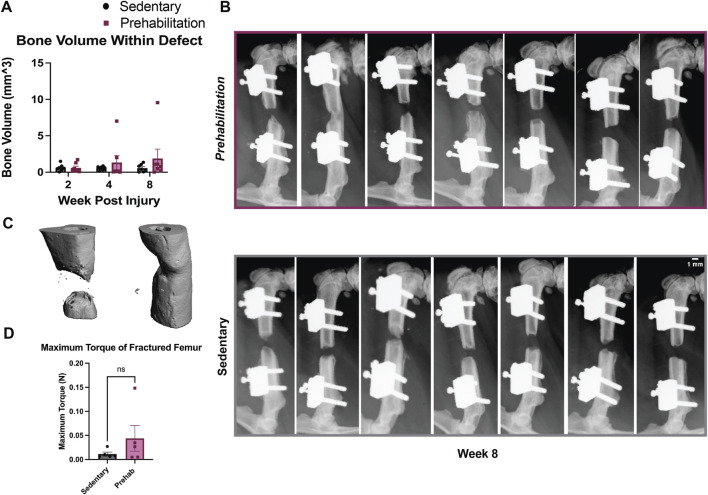
Exercise did not significantly improve regenerated bone volume or bridging **(A)** Longitudinal bone volume quantification within the central 1.5 mm of 2 mm defects. Although not significant, at week 8 post-fracture mean bone volume within the defect region for prehab animals was 3.4x greater than sedentary controls, driven by one subject which bridged. **(B)** Radiographs of all prehabilitation and sedentary animals after 8 weeks of healing. Callus formation was only seen in the outlying prehabilitation subject. **(C)** 3D micro-CT reconstructions of prehab and sedentary subjects in the region between fixation plate ends to demonstrate visually healing vs nonhealing. **(D)** Longitudinal data were analyzed with a repeated measures two-way ANOVA, with multiple comparisons corrected for with Tukey’s post-hoc test (n = 7/group, p < 0.05). Torque data was analyzed with a Mann-Whitney test (n = 5/group, p < 0.05). All data are displayed as mean ± sem.

Regenerated bone volume was assessed longitudinally by *in vivo* micro-computed tomography (µCT). The region of interest used to quantify bone volume post-operatively was the central 1.5 mm of the 2 mm bone defect along the femoral axis. Therefore, bone volume refers only to newly regenerated bone within the defect. No significant differences were observed in the final bone volume *in vivo* or *ex vivo*, or in mechanical properties between the prehabilitation and sedentary group (Figures 3A,D). However, with the prehabilitation group, we observed one subject bridge completely, giving a final bridging rate of 14%, as compared to 0% in the sedentary group (Figure 3B), seen visually via 3D reconstruction (Figure 3C). To ensure the lack of significance was not due to an insufficient sample size, a post-hoc power analysis was run in G*Power software. Given an effect size of F = 1.168 and α err probability 0.05, our actual power was found to be 0.9503, suggesting an n = 7/group was sufficient to capture any statistical significance, indicating prehabilitation was an insufficient intervention to promote bone regeneration.

### 3.3 Prehabilitation attenuated the pain response to fracture

**FIGURE 4:**
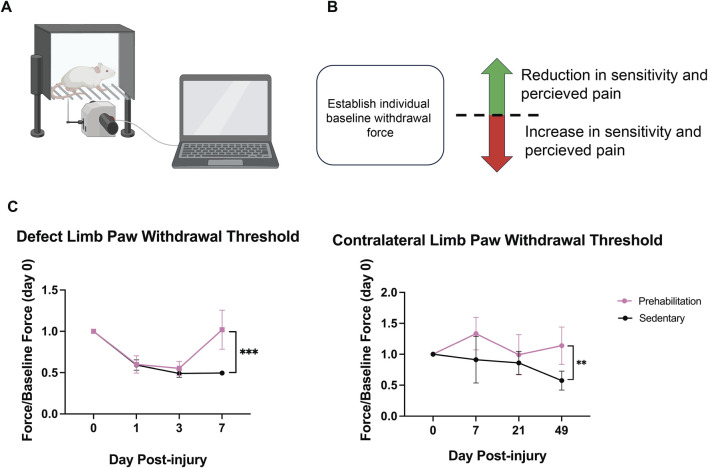
Exercising animals recovered to baseline tactile allodynia **(A)** Graphical representation of Von Frey system with wire mesh bottom, and electronic filament for withdrawal force quantification **(B)** A baseline withdrawal force is established for all subjects. A measured force post-fracture greater than the baseline is perceived as reduction in pain, with the inverse being true for a decreased withdrawal force. **(C)** Longitudinal paw withdrawal force normalized to baseline measurements for injured and uninjured limbs. The exercising group recovered to baseline levels and had a significantly greater withdrawal force than sedentary (***p < 0.0009). Exercising subjects also had a significantly higher contralateral paw withdrawal threshold as compared to sedentary at week 7 (**p < 0.001). Data were analyzed with a mixed-effects model (n = 6–7/group, p < 0.05), with multiple comparisons corrected for with Sidak’s post-hoc test and displayed as mean ± sem.

Nociception was assessed as a metric for longitudinal pain response via electronic Von Frey. Direct measurements can be found in Supplementary Table S2. Pain attenuation is marked by a subject’s paw withdrawal force value returning to baseline, an indication of functional recovery. Whereas, prolonged pain produces a continued lower withdrawal force, remaining significantly below baseline. Baseline paw withdrawal force was comparable between animals in both groups prior to surgical defect creation. In the early weeks (<3 weeks) following surgery, paw withdrawal thresholds in both groups were significantly reduced from baseline (p < 0.006). However, at week 7, the prehabilitation group recovered to baseline levels of paw withdrawal force, whereas the sedentary group’s withdrawal force remained significantly below baseline, and the withdrawal forces were significantly different between the two groups at 7 weeks (Figure 4C). Interestingly, the sedentary subject’s contralateral uninjured limbs also had significantly reduced paw withdrawal threshold as compared to the prehabilitation group (Figure 4C). These data indicate improved pain recovery in the prehabilitation group, despite insignificant effects on bone regeneration.

### 3.4 Prehabilitation did not influence pre-operative immune status, but significantly attenuated the post-fracture systemic immune response

**FIGURE 5:**
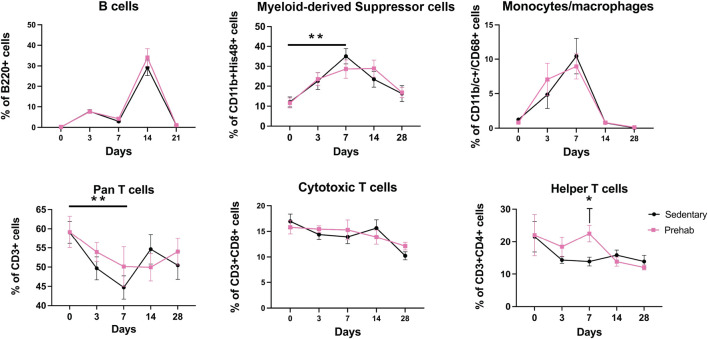
Longitudinal profiling of systemic immune cell populations shows elevated immunosuppression and decreased pro-osteogenic cells in sedentary animals (Top middle) Systemic quantification of CD11b+His48+ myeloid derived suppressor cells revealed elevation of this immunosuppressive population at 1-week post-fracture sedentary subjects only. (Bottom left) CD3^+^ Pan T cells were depressed 1-week post-fracture in only the sedentary subjects. (Bottom right) There were significantly more CD4^+^ Helper T cells in the exercising group at day 7 as compared to the sedentary subjects. Data were analyzed with a mixed-effects model (n = 5–7/group, p < 0.05, *p < 0.05, **p < 0.01), with multiple comparisons corrected for with Sidak’s post-hoc test and displayed as mean ± sem.

Systemic immune cell populations were quantified at baseline (after prehabilitation, but before injury), and at key timepoints during healing. Assessment of these cell populations revealed that at day 7, myeloid-derived suppressor cells (MDSCs) were significantly increased in the sedentary group relative to baseline (Figure 5 (top middle), consistent with other research that utilized bone injury models (Kawai et al., 2021). No significant longitudinal MDSC increase was observed in the prehabilitation subjects. The sedentary group had a significant concomitant longitudinal decrease in T cells at day 7 compared to baseline (Figure 5 (bottom left)). At day 7 animals in the prehabilitation group had significantly more CD4^+^ T cells present (Figure 5 (bottom right)) as compared to the sedentary group. No differences between groups in monocyte/macrophage, or B cell populations were observed at any timepoint (Figure 5). These systemic immune profile differences were not sustained, and by day 14 there were no significant differences in any profiled immune cell types. Overall, in two of the quantified cell types, prehabilitation attenuated longitudinal systemic immune response changes in response to fracture as compared to baseline, and elevated day 7 helper T cells as compared to sedentary.

## 4 Discussion

Despite advances in bone fracture care, non-union rates have remained steady for decades with severe functional and financial consequences for patients (Mills et al., 2017). There are several treatment options for poor healing fractures, however a comprehensive understanding of the mechanisms of non-union remains elusive, making it difficult to predict which fractures will need additional intervention (Evans et al., 2024). In this study we sought to interrogate the influence of an individual’s history of exercise on bone regeneration outcomes following femoral fracture in a rodent model. One group of animals trained for 4-weeks on treadmills, running at 70%–75% of their VO2 max (Mankowski et al., 2024), while another group remained sedentary leading up to surgical femoral defect creation. Sedentary subjects were brought into the treadmill facility and placed on a non-moving treadmill to equilibrate stress of transport and handling. Femurs in both groups were internally fixed with compliant plates and segmental bone defects were created. All animals remained sedentary in the 8 weeks following defect creation. The term sedentary in this case means that animals were not exercised, but in-cage ambulatory activity was permitted and not quantified. All animals received pre-operative μCT scans and healing was monitored via longitudinal radiographs and μCT scans after fracture, as well as endpoint mechanical testing. To investigate the biological mechanisms of healing, we profiled the systemic immune response after fracture via longitudinal blood draws. This study provides an assessment of how a history of exercise, termed “prehabilitation,” may influence bone healing outcomes following fracture, and gives insight into potential immune-mediated mechanisms.

Given the well-known adaptations of bone to mechanical loading and the immune response to aerobic exercise, we hypothesized that running prehabilitation would result in improved bone healing outcomes (Bergmann et al., 2010; Hong and Kim, 2018; Sundh et al., 2018). Unexpectedly, we found that prehabilitation prior to surgical fracture induction did not improve bone’s structural integrity pre-fracture. In fact, it had the opposite effect. Prehabilitation subjects had significantly lower bone properties than their sedentary counterparts after a month of load-bearing exercise. This inhibitory response to load-bearing exercise may have been partially due to the rats’ skeletal immaturity (aged-9-week at initiation of the treadmill protocol) during the exercise period. Several studies have elucidated differences in the response of bone to weight-bearing exercise in adolescent and aged rodents (Bourrin et al., 1994; Gardinier et al., 2018). The impact of exercise-induced skeletal loading on bone structure varies depending on the type of loading (inclined vs decline running, etc.), age, and sex of the animals (Portier et al., 2020). Maturing female rats demonstrated bone adaptations consistent with our results in some, but not all, cases (Bourrin et al., 1994; Oh et al., 2016; Mustafy et al., 2019). In our study, load-bearing exercise appears to have stunted age-associated increase in bone volume, bone mineral density, and both cortical thickness and polar moment of inertia at both the proximal and distal ends of the mid-diaphyseal region of femurs. It is important to note that differences in structural properties of bone did not translate to impaired bone formation after injury or repressed functional recovery. It is possible that the selected exercise protocol may have been too intense for the subjects before reaching skeletal maturity. Although caloric and metabolic data were not collected, all animals had access to food *ad libitum*. These data present an opportunity for future age and sex related studies of bone adaptations to load-bearing exercise.

Despite having decreased bone properties at time of fracture, the prehabilitation animals had 3.4x fold higher bone formation in the fracture defects after 8 weeks compared to sedentary subjects, although this difference was not statistically significant and primarily due to one subject that fully bridged. Post-hoc power analysis revealed that our actual power was 0.9503, indicating our sample size was sufficient to detect effects of prehabilitation and time. The bridging rates we observed in this study in both the prehabilitation, and sedentary group (14% and 0%, respectively) were lower than we would have expected for this model. This low bridging rate in a 2 mm defect stabilized with a compliant fixation plate is below what we have historically seen in our model (Williams et al., 2023), where bridging rates across all our studies are approximately 35%. These subjects were 1 week younger than the standard age at which we have previously induced injury, which may have contributed to the lack of healing, but this requires further investigation.

Although the bone defects did not consistently regenerate bone to bridge the defect, prehabilitation animals demonstrated more rapid pain attenuation. We assessed longitudinal tactile allodynia using electronic Von Frey. Initial pain responses between groups were similar through the first 3 weeks post-fracture. However, we found that by week seven prehabilitation animals had significantly reduced limb sensitivity to stimulation as compared to sedentary controls. At week 7, prehabilitation animals had a normalized paw withdrawal threshold equivalent to their baseline levels, whereas sedentary animals did not return to baseline, indicating sustained pain. These data indicate a trend toward earlier restored function and pain reduction in animals with a history of prior exercise. Research suggests that pre-fracture handgrip strength, commonly accepted as an objective measure of physical function, is significantly related to functional recovery in an elderly population (Savino et al., 2013), suggesting pre-fracture muscular strength may contribute to improved functional outcomes post-fracture, and potentially explaining our observations of decreased tactile allodynia in the prehabilitation group. Other groups have examined the neuroimmune influences of pre-injury exercise on pain sensitivity during recovery and found similarly that mechanical allodynia scores measured with von Frey were significantly better in pre-injury exercise groups as compared to non-exercise controls (Koop et al., 2023).

One of our primary objectives in this study was to assess whether the immune response to injury might contribute to a cellular understanding of how exercise mediates musculoskeletal healing outcomes. Due to the known importance of the immune response in fracture healing, we chose specifically to interrogate the longitudinal systemic immune profile (Lopez et al., 2022). While prehabilitation did not significantly improve bone regeneration, we found that it did result in subtle but significant divergence in the early systemic inflammatory response after fracture between exercising and sedentary subjects. In the week after injury, sedentary subjects had a significant longitudinal increase in MDSC levels, which have been previously associated with reduced bone formation and chronic immunosuppression (Cheng et al., 2021; Vantucci et al., 2022). Prehabilitation animals had significantly greater helper T cells and did not see a significant longitudinal increase in MDSC levels 1-week post-fracture, unlike sedentary subjects. These differences suggest a modest positive effect of pre-injury exercise on the post-fracture immune response. Helper T cells in particular, have been shown to be pro-osteogenic and can contribute to bone regeneration (Maruyama et al., 2020), and are characteristic of a pro-regenerative early systemic and local inflammatory profile associated with regular pre-injury exercise (Schmidt-Bleek et al., 2012; Lord et al., 2014; Lopez et al., 2022). By 14 days post-injury there were no differences in the systemic cellular immune profiles between sedentary and prehabilitation groups. Recent studies in mouse models have shown metabolic alterations in macrophages for only 2 weeks after exercise, suggesting a limited chronic effect of exercise on immune populations, which may provide an explanation for the duration of the pro-regenerative profile seen in the prehabilitation group (Murugathasan et al., 2023). These results are promising and demonstrate a potential mechanism through which a history of regular exercise may influence musculoskeletal healing outcomes.

In this study, there are several limitations of note including (1) the inclusion of only female animals (2) the young age of the rats at the start of prehabilitation and time of surgery (3) only one prescribed exercise protocol of high-intensity, (4) a relatively small panel of profiled immune populations, and (5) a defect which was critically-sized in this model. Historically, we have used female rats due to complications which arise in the male rodent population such as rapid skeletal growth and weight gain which would occur over the 8-week experimental period. Weight gain causes several issues which prohibit the use of male rats in studies with longitudinal imaging. Primarily, their large size makes it difficult to accommodate their limbs during *in vivo* micro-CT imaging to quantify bone regeneration, and excessive loading may cause plate hardware failure. It is known there are sex-based differences in response to exercise (Berro et al., 2024), for example, female athletes clinically experience a higher rate of bone stress fractures (Chen et al., 2013). Here, we observed detrimental effects of load-bearing exercise on growth in our skeletally immature female rodents, but future work will be needed to assess whether these results are generalizable to both sexes.

This ties in with our second limitation, that all rodents were skeletally immature at the time of prehabilitation onset. In our rat segmental defect model, we typically perform surgery on animals between 13 and 15 weeks of age when their skeletal growth plateaus. For this study we began exercise at age 9-weeks, so that animals would be 14 weeks of age at the time of surgery in accordance with past work. The unanticipated suppressive effects of exercise on skeletal growth introduce a potential confounding factor when assessing the effects of exercise on bone healing. It is possible that the observed negative effects on growth affected the healing response to mechanical stimulation within the defect, and concealed the effects of prehabilitation on bone regeneration, creating a superposition of factors. As such, the relevance of our results is limited to how exercise may affect bone healing in growing adolescent females, and we cannot generalize our conclusions to skeletally mature animals. To address this limitation, future work should begin the prehabilitation protocol after 14 weeks to determine if prehabilitation is a sufficient intervention in a skeletally mature population.

Third, we chose a high-intensity exercise protocol based on the Molecular Transducers of Physical Activity Consortium (MoTrPAC (Sanford et al., 2020)). High-intensity aerobic exercise has been shown to have a greater impact on the systemic immune profile, however high strains can be deleterious to bone and a moderate exercise group may have improved regenerative outcomes (Jonsdottir and Hoffmann, 2000; Schmal et al., 2020). Fourth, the developed flow cytometry panel for this study examined only a small subset of immune cells. As the fields of exercise immunology and osteoimmunology grow, additional immune cell subtypes have been identified as key components of the immune response and bone maintenance and regeneration (Schlundt et al., 2024). Flow cytometry for this study was conducted on a two-laser cytometer in which we created three panels. Anti-rat antibodies are expanding in prevalence and in future studies we plan to work with a 5-laser cytometer and expand our immune panel to capture additional populations. Lastly, we opted to use a defect size that previously is on the threshold of being critically-sized to provide room for potential improvement by prehabilitation treatment. It is possible that a less severe, non-critically-sized bone injury may have yielded different results. Future studies are needed to understand whether injury severity level affects the bone healing response to exercise.

Overall, this study showed that for skeletally immature female rats a history of exercise prior to bone fracture modulates the early immune response to fracture and improves post-injury pain sensitivity. However, the modest positive effects on the immune response were not sufficient to orchestrate significantly improved healing or bridging rates without additional intervention. Prior work in our group revealed improved bone healing with exercise re-introduced 1 week after injury (Klosterhoff et al., 2020; Williams et al., 2024). Taken together, these studies imply that a combination of pre- and post-surgical exercise protocols may beneficially modulate immune response to injury and promote functional regeneration. This work has translational implications for developing data-driven fracture management plans for young athletes who suffer fractures. A combination of CT and immune data may be indicative of an individual’s likelihood to heal and can be incorporated into a return-to-play plan. Further, these data have the potential to be applied to groups which are likely to undergo surgeries which result in bone loss. For example, in adolescents, developmental dysplasia of the hip is treated surgically with a periacetabular osteotomy, wherein a prehabilitation regime may help with functional recovery and bone regeneration (Kołodziejczyk et al., 2022). Other examples of known reductions in bone health include childhood cancer treatments, where fractures in adulthood may have conditions similar to this model (Wasilewski-Masker et al., 2008). Future work will continue profiling the early immune response to exercise in a rehabilitation-mediated healing model to prospectively identify fractures at-risk for delayed or failed healing.

## Data Availability

The raw data supporting the conclusions of this article will be made available by the authors, without undue reservation.
