# Supplementary material for: Load-bearing aerobic exercise prior to injury moderates systemic immunosuppression response to fracture
Source: Front Physiol. 2025 Sep 11;16:1587766. doi: 10.3389/fphys.2025.1587766 (PMC12460303; doi:10.3389/fphys.2025.1587766)
Supplement: Supplementary file 1 [file Table1.docx]

| **Target** | **Reactivity** | **Fluorophore** | **Clone** | **Supplier** | **Dilution** | **Concentration** |
| --- | --- | --- | --- | --- | --- | --- |
| His48 | Rat | FITC | His48 | eBioscience | 1:25 | 0.5 mg/ml |
| CD11b/c | Rat | PE | OX-42 | eBioscience | 1:25 | 0.2 mg/ml |
| CD68 | Rat | FITC | ED1 | eBioscience | 1:25 | 0.1 mg/ml |
| CD4 | Rat | APC | OX35 | eBioscience | 1:25 | 0.2 mg/ml |
| CD3 | Rat | PE | eBioG4 | eBioscience | 1:25 | 0.2 mg/ml |
| CD8 | Rat | PE-Cy7 | OX8 | eBioscience | 1:25 | 0.2 mg/ml |
| CD86 | Rat | PE | 24F | eBioscience | 1:25 | 0.2 mg/ml |
| CD163 | Rat | AF 647 | ED2 | Bio-Rad | 1:25 | 0.05 mg/ml |
| B220 | Rat | PE-Cy7 | His24 | eBioscience | 1:25 | 0.2 mg/ml |
| CD11b/c | Rat | PerCP | OX-42 | Novus | 1:25 | 0.2 mg/ml |

**Table S1: Antibodies for the three flow cytometry panels used.** Analysis was performed on the BD accuri C6 Plus. The cytometers two lasers and four channels permit for four stains at a time. Multiple conjugates were required in some cases, as in CD11b/c.
