# Supplementary figures and images for: Load-bearing aerobic exercise prior to injury moderates systemic immunosuppression response to fracture
Source: Front Physiol. 2025 Sep 11;16:1587766. doi: 10.3389/fphys.2025.1587766 (PMC12460303; doi:10.3389/fphys.2025.1587766)

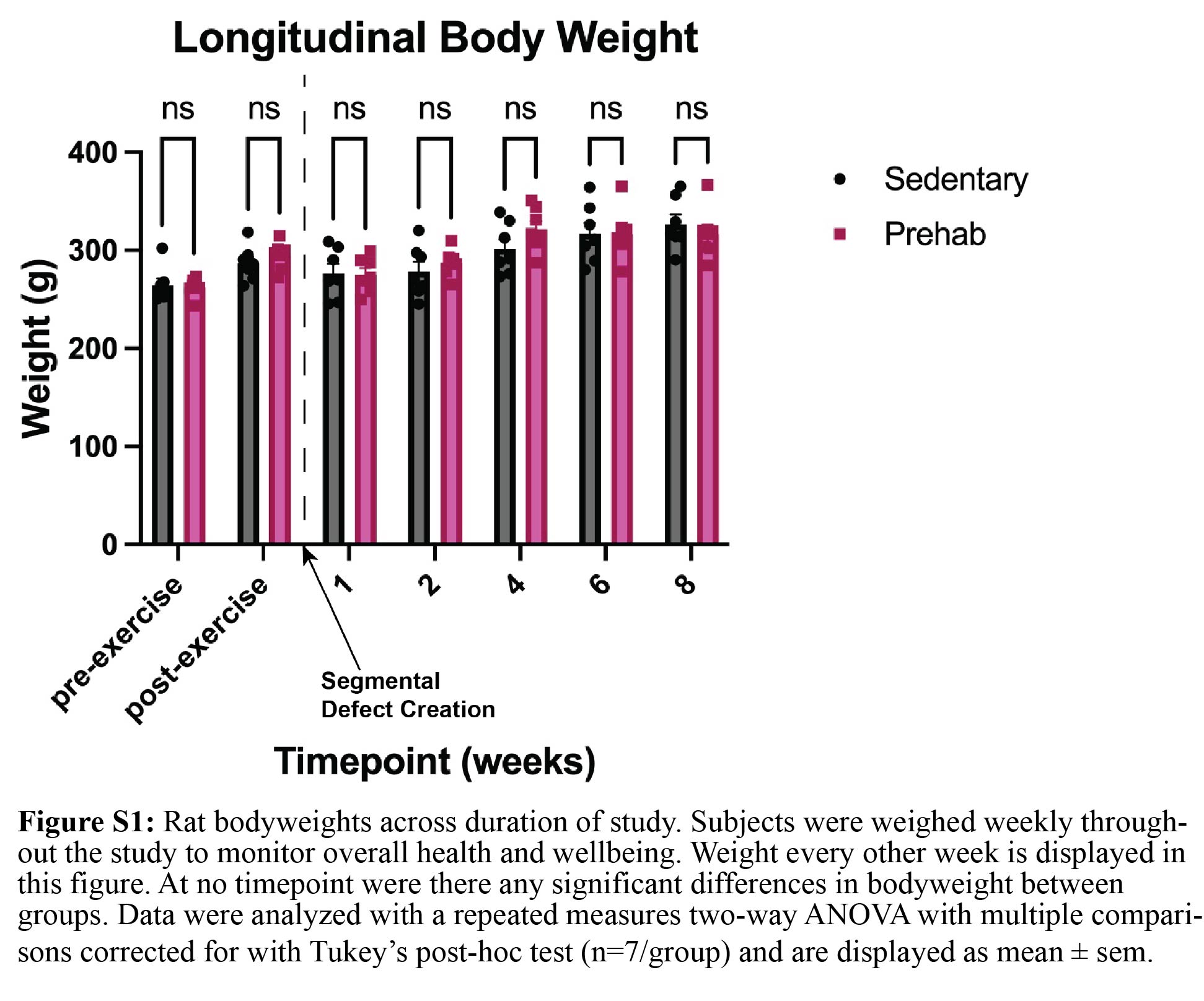

Supplement: Supplementary file 2 [file Image1.jpeg]

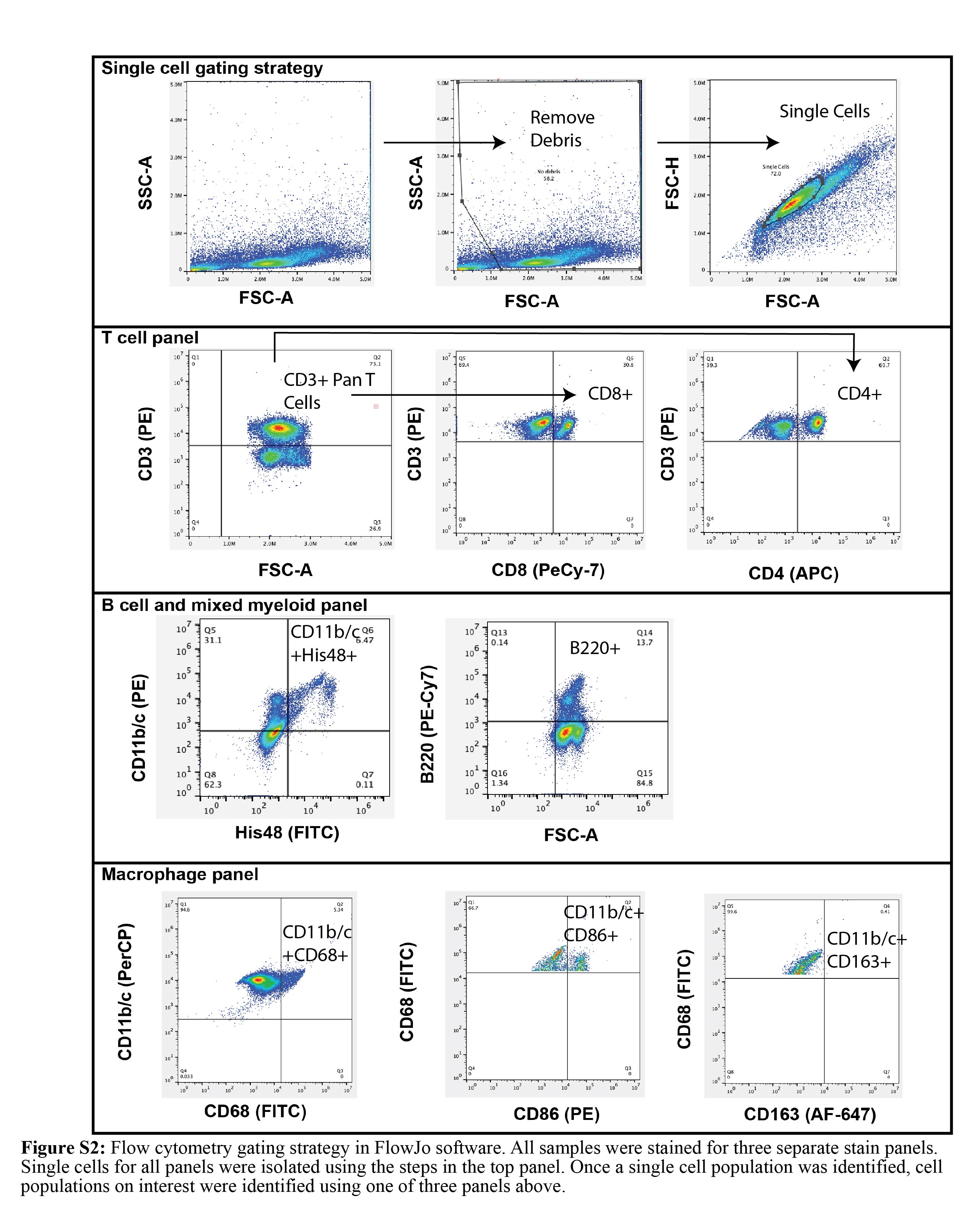

Supplement: Supplementary file 3 [file Image2.jpeg]
