# Supplementary material for: Load-bearing aerobic exercise prior to injury moderates systemic immunosuppression response to fracture
Source: Front Physiol. 2025 Sep 11;16:1587766. doi: 10.3389/fphys.2025.1587766 (PMC12460303; doi:10.3389/fphys.2025.1587766)

**Table S2: Raw Von Frey Data.** All animals had filaments applied to both limbs in three separate trials. An average of these trials was then computed, and that number taken as the subject’s paw withdrawal threshold for the timepoint. For timepoint after baseline, an additional row for normalization of the average withdrawal force value to value exists.


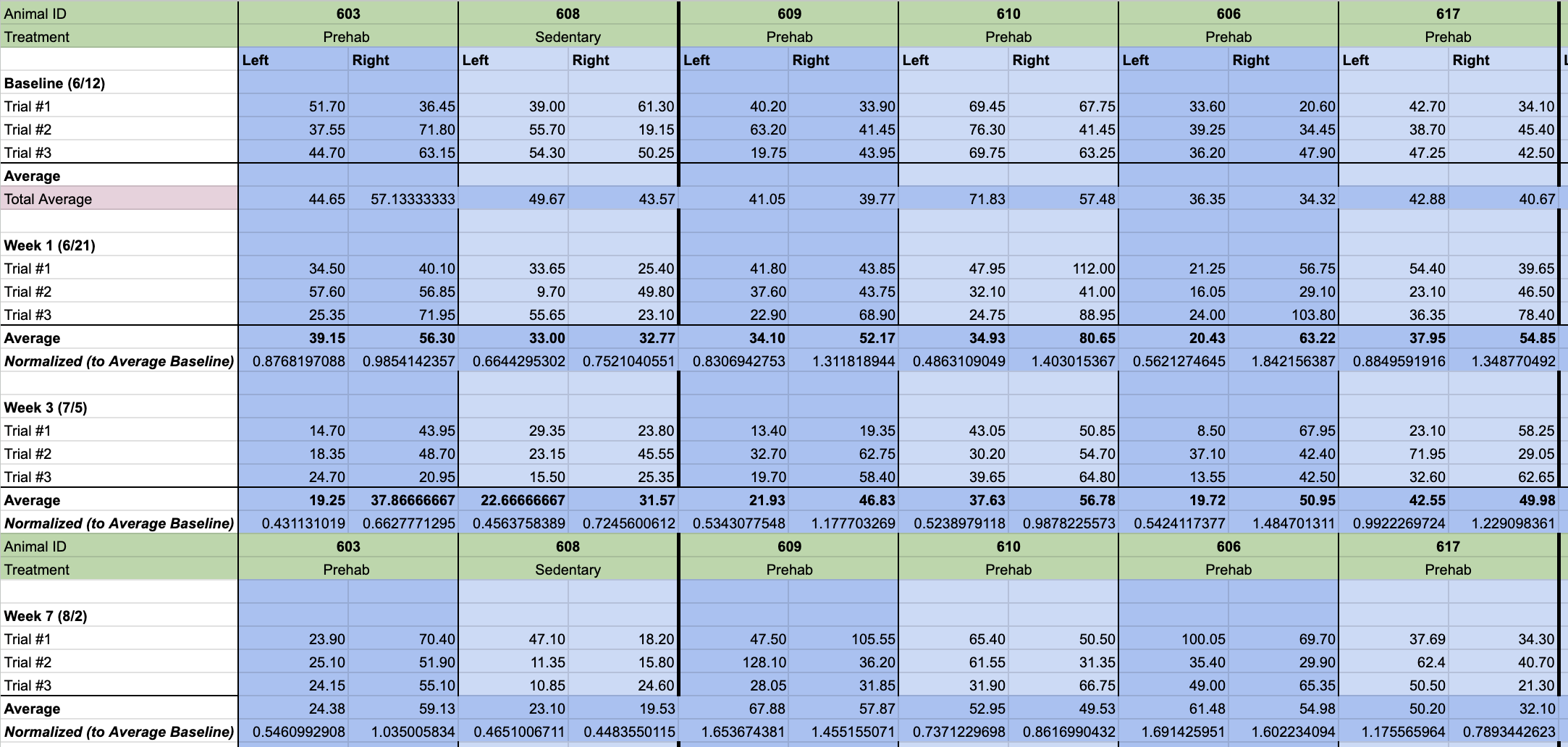


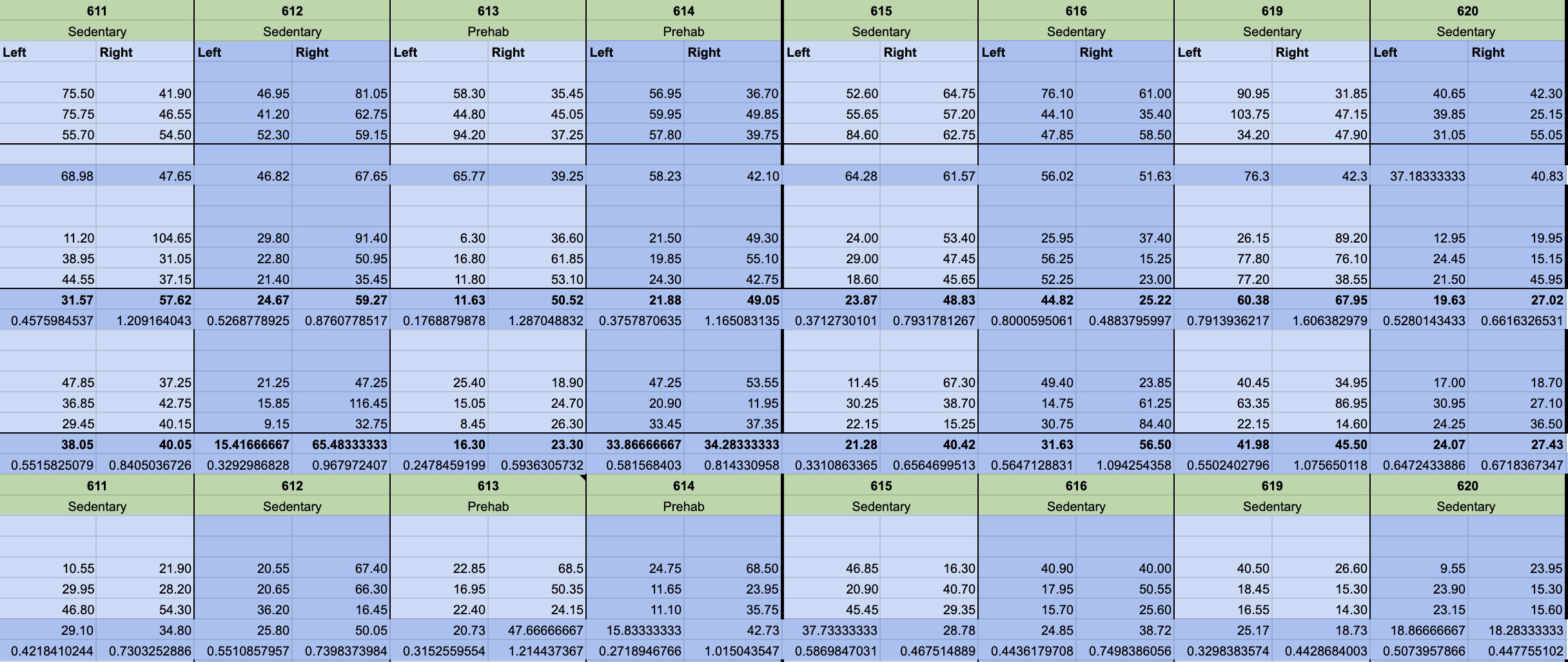

Supplement: Supplementary file 4 [file Table2.docx]
